# A Case of Pleomorphic Dermal Sarcoma: Giant Exophytic Tumor of the Medial Canthus

**DOI:** 10.3390/dermatopathology11010003

**Published:** 2023-12-29

**Authors:** Rylee Moody, Kavita Darji, Tricia A. Missall, Peter Chow, Ramona Behshad

**Affiliations:** 1 School of Medicine, Saint Louis University, 1008 S Spring Ave, St. Louis, MO 63110, USA; 2 Department of Dermatology, SSMHealth SLUCare, Saint Louis University, 1008 S Spring Ave, St. Louis, MO 63110, USA; 3 Department of Dermatology, University of Florida, Gainesville, FL 32606, USA

**Keywords:** pleomorphic dermal sarcoma, atypical fibroxanthoma, pleomorphic leiomyosarcoma, and keratoacanthoma, dermatopathology

## Abstract

We present the case of a 99-year-old Caucasian female who was referred for treatment of a painless, 8.0 cm × 7.8 cm exophytic, pedunculated, ulcerated tumor of the left medial canthus. Pathology showed spindled, oval, and polygonal cells with pleomorphic nuclei. Many multinuclear giant cells and mitotic figures were also noted. The tumor was highlighted with CD10, showed focal positivity with actin, desmin, and CD68, and had increased Ki67 immunohistochemical staining. The tumor was negative for pancytokeratin, CK5/6, p63, MART-1/MelanA, S100, Sox10, p40, CD34, and CD23. Based on clinicopathologic correlation, the diagnosis of pleomorphic dermal sarcoma (PDS) was made. Pleomorphic dermal sarcoma (PDS) refers to a deep, histologically high-grade tumor that often resembles other tumors clinically and histologically. As PDS is frequently aggressive and related to adverse outcomes, it is important to recognize its distinguishing features in comparison to other similar entities, including atypical fibroxanthoma (AFX) and pleomorphic leiomyosarcoma (PLMS). To our knowledge, there is only one other reported case in the literature of PDS occurring on the eye. By reviewing and understanding characteristic etiologies, locations of presentation, histopathological features, and management techniques, pathologists can make a more accurate diagnosis and dermatologists can provide more effective patient care in a timely manner.

## 1. Introduction

Pleomorphic dermal sarcoma (PDS) is a histologically high-grade tumor that can often resemble other tumors both clinically and histologically [1]. As PDS is frequently aggressive and related to adverse outcomes [2], it is critical to recognize its distinguishing features in comparison to similar entities. Differential diagnoses of PDS include atypical fibroxanthoma (AFX) and pleomorphic leiomyosarcoma (PLMS) [1,2,3,4]. Hereafter, we describe the case of a patient who presented with an extensive tumor on the face that was eventually diagnosed as PDS. To our knowledge, there is only one other reported case in the literature of PDS occurring on the eye [5], making this an extremely rare presentation of the disease.

## 2. Case Presentation

**Figure 1:**
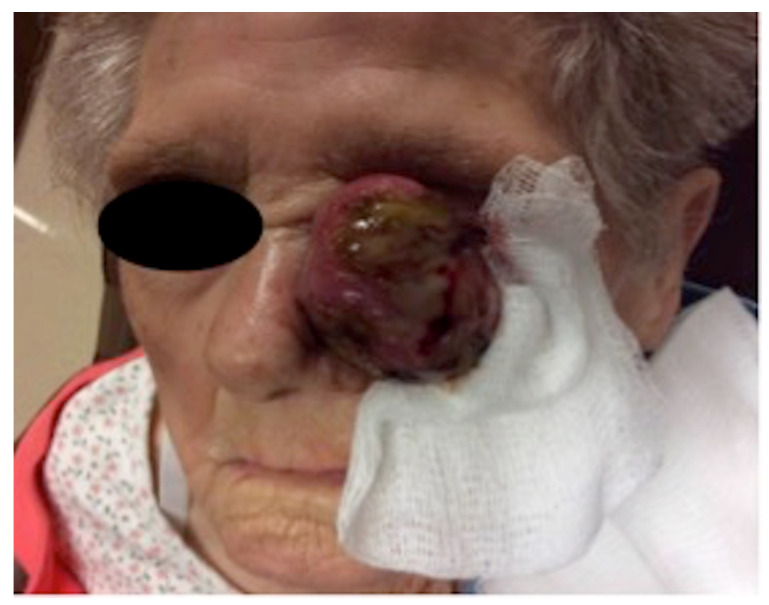
Large, exophytic, ulcerated tumor of the medial canthus of the left eye at the initial consultation.

A 99-year-old Caucasian female was referred for treatment of a large, painless tumor on her left medial canthus. Ophthalmic history was unremarkable, with no history of antecedent trauma or radiation. It had been growing slowly for one year, followed by rapid growth after superficial biopsy by her outside dermatologist. At the consultation, the patient had an 8.0 cm × 7.8 cm exophytic, pedunculated, ulcerated tumor of the left medial canthus (Figure 1). It was firm, non-pulsatile, and fixed to the underlying tissue. There was no lymphadenopathy of the head or neck.

**Figure 2:**
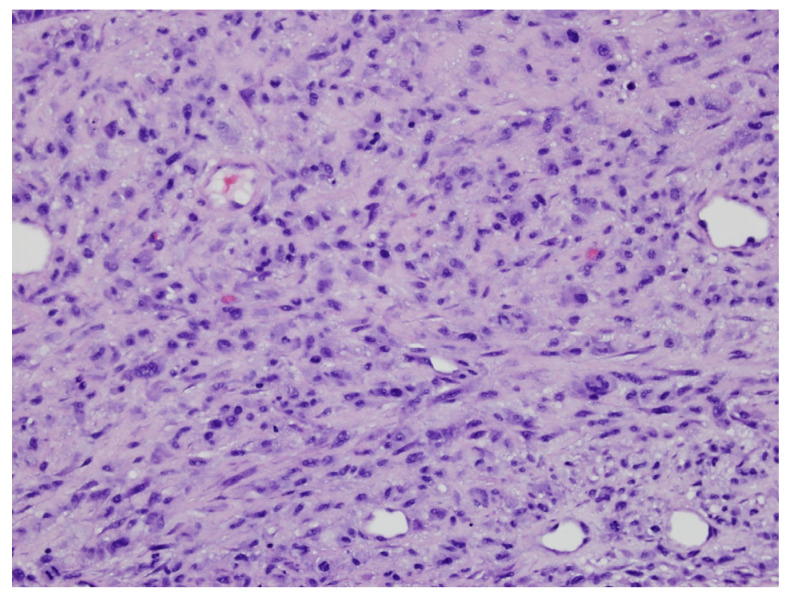
Spindled, oval, and polygonal cells with pleomorphic and polymorphic nuclei (H&E original magnification ×200).

**Figure 3:**
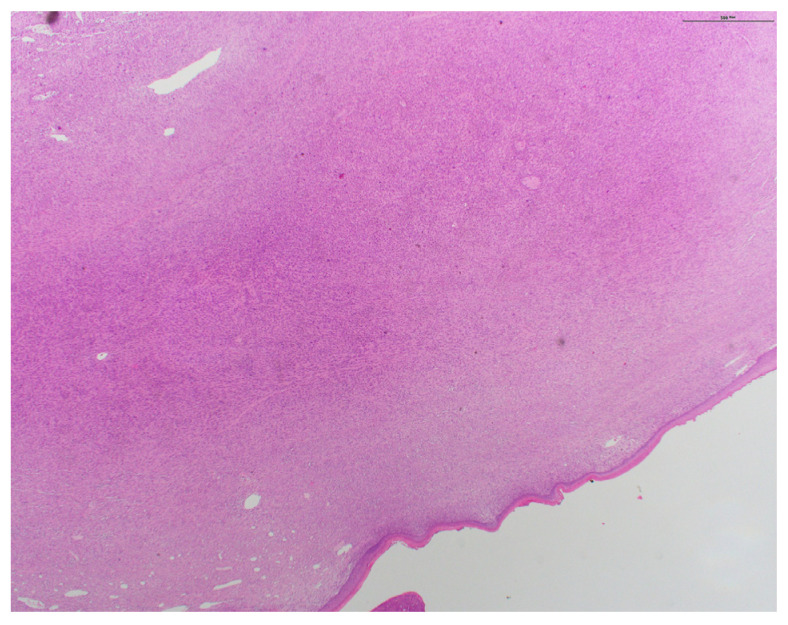
Diffuse dermal spindle cell proliferation with spindled, oval, and polygonal cells (H&E original magnification ×20).

**Figure 4:**
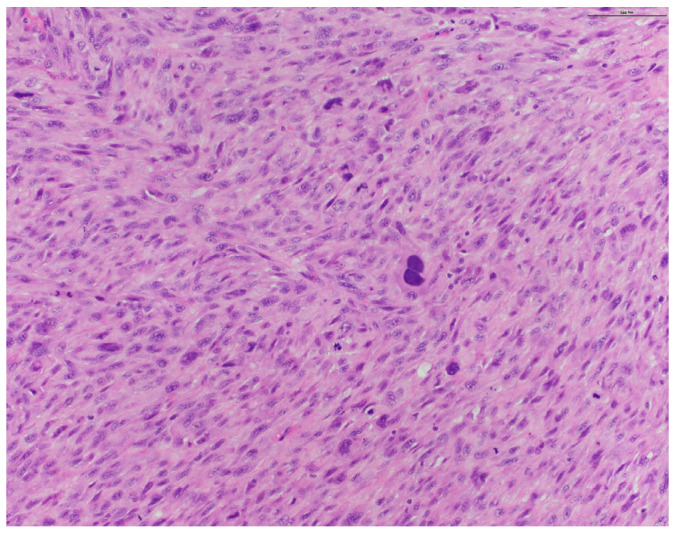
Higher magnification highlighting many multinuclear giant cells and mitotic figures (H&E original magnification ×200).

**Figure 5:**
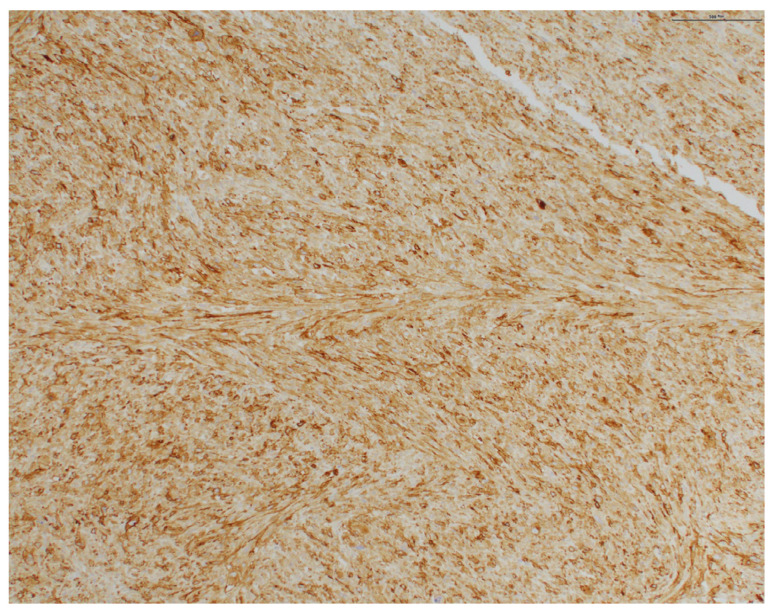
Immunohistochemical staining showing increased CD10 staining (original magnification ×100).

Pathology from subsequent staged Mohs excision showed diffuse dermal spindle cell proliferation. There were spindled, oval, and polygonal cells with pleomorphic nuclei (Figure 2 and Figure 3). Many multinuclear giant cells and mitotic figures were also noted (Figure 4). No necrosis or vascular or perineural invasion was seen. The tumor was highlighted with CD10 and showed focal positivity with actin, desmin, and CD68 (Figure 5). There was increased Ki67 immunohistochemical staining. The tumor was negative for pancytokeratin, CK5/6, p63, MART-1/MelanA, S100, Sox10, p40, CD34, and CD23 imunohistochemical staining. The histologic differential diagnosis included AFX, PDS, and PLMS. Clear margins were obtained after three staged excisions, with the defect extending to the bone. On the basis of these findings, the diagnosis was revised to PDS.

## 3. Discussion

The original nomenclature used to describe tumors resembling PDS included the terms ‘superficial malignant fibrous histiocytoma’ and ‘undifferentiated pleomorphic sarcoma of skin’, which were first described by Joseph O’Brien in 1964 [6,7]. However, it was not until 2012 that the paradigm shifted, embracing the terminology of PDS under Professor C. D. Fletcher [7]. This pivotal shift in terminology was motivated by the importance of distinguishing the more concerning histologic features exhibited by PDS, compared to the more superficial and benign character of AFX. One year later in 2013, PDS was recognized as a distinct diagnostic entity by the World Health Organization Classification of Soft Tissue Tumors, and the term ‘superficial malignant fibrous histiocytoma’ was discarded [7].

PDS is a rare, aggressive tumor that is not well documented in the literature, although it is the most commonly diagnosed soft tissue sarcoma in the head and neck [2,8]. For this reason, it is crucial to make an accurate diagnosis. AFX and PDS represent a tumor spectrum with overlapping clinical and histologic features. Because of their similar characteristics, it is vital to appreciate their distinguishing factors for accurate diagnosis and subsequent therapy and prognosis. AFXs usually present as superficial, rapidly growing papules or nodules, which may ulcerate or bleed [1]. Similarly, PDSs usually present as rapidly growing and frequently ulcerating tumors that show nodular, polypoid, or, less frequently, plaque-like growth patterns. Both tumors arise in sun-damaged skin, predominately in older, Caucasian males [2].

Histologically, PDSs can display deeper subcutaneous extension, lymphovascular invasion, perineural invasion, and necrosis, which may help distinguish them from the more superficial AFXs [1]. Immunohistochemically, PDSs and AFXs usually stain negative for cytokeratins, S100, HMB-45, MelanA, desmin, h-caldesmon, and CD34. Focal expression of epithelial membrane antigen has been observed in approximately 15% of cases. Focal staining for CD31 and smooth muscle actin has been observed in approximately 50% and 70% of cases, respectively. PDSs and AFXs commonly stain positive for CD10, CD99, and vimentin [2,3].

A recent study by Janz et al. evaluated the survival outcomes of patients with head and neck PDSs stratified by grade [8]. The overall survival significantly decreased in a stepwise fashion with each grade. The 5-year overall survival was 54%, with grade I having 69% 5-year overall survival, while grade IV had 42% 5-year overall survival [8]. Given the difference in survival outcomes affected by histological grade, this further emphasizes the significance of proper diagnosis, which requires an adequate biopsy specimen that should contain subcutaneous tissue, regardless of an exophytic appearance, to help distinguish between AFX and PDS, and to determine histologic grade, as this could change the counseling given to patients [8].

Surgery is the gold standard for the treatment of AFX and PDS. While surgical excision is the predominant choice for the majority of PDS cases, local recurrences still occur in up to 28% of patients, predominantly within the first two years subsequent to excision [9]. In stark contrast, AFX tends to have a lower likelihood of recurrence, with it seen in fewer than 5% of cases. Up to 20% of PDSs will metastasize, mainly in the skin, lymph nodes, and lungs, particularly in patients with underlying hemato-oncologic diseases [9]. There is limited research comparing the best excision technique for PDS. However, a study comparing the recurrence and survival rates between Mohs micrographic surgery and wide local excision for AFX found lower recurrence rates in those who received Mohs micrographic surgery [10,11]. It is important to note that those with larger and more aggressive tumors often undergo wide local excision, which may contribute to the observed differences. There is no published data on the sensitivity of radiation for AFX and PDS, though this treatment may be necessary if complete excision is not possible [9]. More research is needed to determine if Mohs micrographic surgery, wide local excision, and adjuvant radiation provide better outcomes for PDS, specifically, given its more aggressive nature [2,3].

Recent studies have shown that PDSs are tumors with a high mutational burden, exhibiting signatures 7a and 7b in almost equal proportions [9]. This contrasts with other UV-induced tumors such as cutaneous squamous cell carcinoma, basal cell carcinoma, and melanoma, which predominately exhibit 7a. They are also very inflammatory and immunogenic tumors, expressing high numbers of CD8+ tumor-infiltrating lymphocytes (TILs) and checkpoint molecule expression including PD-L1, LAG-3, and TIGIT [9]. Pembrolizumab, an anti-PD-1 inhibitor, has been shown to be efficacious in a few PDS cases [9]. This suggests that immune checkpoint inhibitors could be another treatment option for those who are not strong surgical candidates. In these individualized cases, an interdisciplinary approach should be utilized to determine the best course of treatment.

Clinically, in contrast to AFXs and PDSs, PLMSs are usually found on extensor surfaces of extremities rather than the head or trunk. These tumors are also more often seen in males, and the average age of presentation is in the sixth decade [4]. Histologically, PLMSs usually consist of tumor cells that blend with collagenous stroma at the periphery, and there is often a superficial grenz zone present. PLMSs will have myofilaments highlighted by Masson trichrome stains, and reticulin stains will demonstrate a fine reticulin network between fibers. These tumors commonly stain positive for vimentin, smooth muscle actin, h-caldesmon, and pan-muscle actin (HHF35). Approximately 70% of tumors will stain for desmin. Cytokeratin, CD117, S100, and factor XIIIa staining have also been demonstrated in a few cases [4].

While the initial superficial biopsy of the tumor in our case suggested an AFX, the remaining unexamined, large, pedunculated tumor showed substantial subcutaneous involvement consistent with PDS. This case highlights the importance of palpating the tumor and its surrounding tissue prior to planning a diagnostic and/or therapeutic procedure. To the best of our knowledge, there is only one other reported case of PDS occurring in the periocular region, on the lateral canthus [5]. In this case, the tumor was also identified as AFX on the initial biopsy. The patient subsequently underwent Mohs micrographic surgery, and the tumor was found to have invaded the skeletal muscle, revising the diagnosis to PDS. She healed via secondary intention without complications. This case, similar to ours, emphasizes the importance of diagnostic biopsy and complete excision when either AFX or PDS is on the differential.

## 4. Conclusion

Due to the aggressive nature of PDS, it is necessary to diagnose it early and accurately. Although PDS may present analogously to the aforementioned tumors, there are fundamental differentiating characteristics to discern it from the others. By reviewing and understanding characteristic etiologies, locations of presentation, histopathological features, and management techniques, dermatologists can provide more effective patient care and recognize adverse outcomes in a timely manner.

## Data Availability

Data are contained within the article. The data presented in this study are available on request from the corresponding author.
